# Climate change and health: Research challenges for health in the developing countries

**DOI:** 10.4103/0019-5278.72239

**Published:** 2010-08

**Authors:** Harshal T. Pandve

**Affiliations:** Department of Community Medicine, Smt. Kashibai Navale Medical College, Narhe, Pune - 411 041, India

**Keywords:** Challenges, climate change, developing countries, health, research

## Abstract

Climate change has emerged as one of the most important environmental issues ever to confront humanity. Recent events have emphatically demonstrated our growing vulnerability to climate change, and health hazards are a major concern. Research pertaining to the effects of climate change on human health is the need of the hour. This paper discusses the broad challenges in health research in developing countries with specific reference to climate change.

## INTRODUCTION

Weather and climate have been known to affect human health since the time of Hippocrates.[1] Climate change impacts range from affecting agriculture – further endangering food security, sea-level rise and the accelerated erosion of coastal zones – increasing the intensity of natural disasters, species extinction and the spread of vector-borne diseases.[2] The fourth assessment report of the Intergovernmental Panel on Climate Change (IPCC) concluded that adverse health effects are much more likely in low-income countries and vulnerable subpopulations.[3] These disparities may well increase in the coming decades not only because of regional differences in the intensity of environmental changes (such as water shortages and soil erosion) but also because of exacerbations of differentials in economic conditions, levels of social and human capital, political power and local environmental dependency.[4]

## MONEY MATTERS

### Budgeting for health (overall)

In many low-income countries, the health sector is a neglected one. The big question is, “What is the right amount for a country to spend?” or “How much of a nation’s gross national product (GNP) or gross domestic product (GDP) should be devoted to health care?”

In 1981, an indicator, “the number of countries with at least 5% of GNP spent on health,” was proposed for the purpose of monitoring and evaluating the global strategy for health for all by the year 2000 (HFA2000).[5] A recent International Monetary Fund (IMF) study suggested that effective health coverage would require around 12% of GNP in low-income countries in order to meet the international development goals.[6] An appropriate percentage benchmark or target for health spending, like the fictitious target above, is extremely difficult to set. Research is under way to better define the minimum amounts of finance that countries should invest in order to optimally develop their health systems. In its 2001 report, the World Health Organization (WHO)-CMH recommended that the low-income countries should increase their domestic spending on health by an additional 1% of GNP by 2007, and by an additional 2% by 2015, keeping in view the existing and future trends of economic growth.[7]

### Budgeting for health research

Health research is essential for the preparation and implementation of national health policies, for planning health actions and for effective delivery of health services. A quality research requires money. Appropriate financing is critical for this entire process. The biggest question is whether there is enough money for research? For the developed countries, the answer may be yes, but what about the low-income countries?

Each year, more than US $100 billion is spent on health research and development (R and D) by the public and private sectors, which makes health second only to military research in terms of global spending on research.[8] Despite this investment, deficiencies in the process of establishing and supporting a set of priorities for health R and D have led to a situation in which <10% of the public and private financial resources destined for research are devoted to the health problems of 90% of the world’s population, an imbalance known as the “10/90 gap.” This gap has a high economic and social cost, aggravated by the fact that even the 10% that is available is not being used in areas where its impact on health would be the greatest.[9] As a general reference point, the Commission on Health Research for Development (COHRED) and the WHO recommend that governments of developing countries apportion at least 2% of the national health expenditures to health R and D. One issue that is important for a full appraisal of the imbalance between the financial resources allocated to health R and D and the resources aimed at overcoming priority health problems is the absence of systematic monitoring of global spending on this type of research. There is no continuous, reliable and accessible source of information on world spending on health R and D. Consequently, there are no verified quantitative estimates of the resources allocated for research on the principal diseases or risk factors. There is also no consolidated information on the results, products and impacts of these investments on health status.[10]

Basically, the budgetary allocation to the health sector is inadequate in most of the low-income countries and, due to this, overall allocation to health research that is obtained from the budgetary allocation to the health sector is no doubt negligible. Most importantly, the overall health sector budgetary allocations must be increased in view of the health hazards due to climate change. The health research should get a separate budgetary head rather than including it into health budget. This is one of the basic challenges as strong political will and political commitment as well as good governance are required for increasing the investment in health.

## LACUNAE IN SURVEILLANCE SYSTEMS

Surveillance is often defined as the systematic collection, analysis and interpretation of health data and the timely dissemination of these data to policymakers and others. Better surveillance at the country level provides better health information and, thus, better opportunities for countries to improve the health of their citizens. Surveillance offers a systematic approach to data collection and is crucial in helping countries monitor and evaluate emerging patterns and trends of disease. By using health data, governments can formulate policies and programs to prevent disease and to measure the progress, impact and efficacy of preventive efforts already in operation. It is a fundamental tool of public health.[11] Still, most of the developing nations are lacking in the basic monitoring and surveillance system. The surveillance system depends on many factors. These are trained and skilled manpower, the proper and effective channel of information flow and an effective feed-back system. Basically, it is the chain, but this chain is not properly maintained most of the time due to the lack of a basic infrastructure to maintain the surveillance system.

Climate change is a major environmental factor that potentially could lead to the emergence of infectious diseases. The health experts from the Wildlife Conservation Society released a report on October 2008 called “Deadly Dozen: Wildlife diseases in the age of climate change.” This report lists 12 pathogens that could spread into new regions as a result of climate change. All have potential impacts to human and wildlife health as well as global economies. As per the report, monitoring of wildlife health provides us with a sensitive and quantitative means of detecting changes in the environment.[12] Extreme climate events are expected to become more frequent in the coming years with climate change. Recognition of the existence of the problem is the first step toward developing a solution. It is important that the country have climate change and extreme health events due to it on its surveillance system. For this purpose, the surveillance system at the place must be well equipped and well maintained. In surveillance systems, active participation of the private sector is also essential at most of the places, but the private sector is not actively participating or is totally unaware about the surveillance system. Involving private sector in routine surveillance systems is a challengeable task. Lack of an effective surveillance system is one of the biggest challenges for health research.

## LACK OF A COLLABORATIVE APPROACH

Climate change is multifactor issue, and its health effects are also different at different places. At few places, the major health hazard may be due to the rising temperatures, whereas at other places it may be due to floods or drought or may be due to vector-borne diseases. Research so far has mostly focused on the thermal stress extreme weather events and infectious diseases, with some attention to estimates of future regional food yield and hunger prevalence.[13] The WHO is also initiating a campaign against climate change. In 2008, the World Health Day focused on the need to protect health from the adverse effects of climate change. The theme “Protecting Health from Climate Change” puts health at the center of the global dialog about climate change. The WHO selected this theme in recognition that climate change is posing ever-growing threats to global public health safety. Through increased collaboration, the global community will be better prepared to cope with climate-related health challenges worldwide.[14] A broader approach is required for the research, which should be a cross-sector, interdisciplinary collaborative one involving different sectors like weather technology, vector bionomics, entomology, architecture, agriculture and food supply, pharmaceuticals, renewable energy sector, automobile and transport sectors, water management and supply sector, pollution control sectors and disaster management. This is one of the greatest challenges in research in many countries to coordinate the different sectors. It is necessary to establish a separate “Department for the Collaborative Research in Climate Change,” with apex bodies for operational research and training at various levels in all the countries. Efforts must be taken to sensitize the general population about the climate change.[15] To spread the awareness and disseminate knowledge in the general population to change their day-to-day activities is another challenge. It is essential for the health policy planners and administrators to consider climate change as a major public health problem in the near future and plan accordingly.[16]

To conclude, the major challenges in the health research in developing countries are inadequate budgetary allocation to health sector and health research, deficiencies in surveillance system, lack of a cross-sector, interdisciplinary collaborative approach in research and lack of awareness about the climate change and its health hazards among the general populations.

## References

[1] Hippocrates, Lloyd GE (1978). Airs, waters and places. An essay on the influence of climate, water supply and situation on health. Hippocratic Writings.

[2] Pandve HT (2008). Global initiatives to prevent climate change. Indian J Occup Environ Med.

[3] (2007). Intergovernmental Panel on Climate Change. Climate change 2006: impacts, adaptation and vulnerability.

[4] Corvalan C, Hales S, McMichael AJ (2005). Ecosystems and human wellbeing: health synthesis.

[5] (2000). WHO, Health for all 2000 (HFA2000) Series No. 4, Development of indicators for monitoring and evaluation of HFA2000, and Health for all 2000 Series No.3, Global strategy for Health for All by the Year.

[6] Gupta S, Verhoeven M, Tiongson ER (2001). Public spending on health care and the poor.

[7] (2001). WHO, Report of the Commission on Macroeconomics and Health, Macroeconomics and Health: Investing in Health for Economic Development (WHO-CMH).

[8] (2004). Global Forum for Health Research. Monitoring financial flows for health research.

[9] (2002). Global Forum for Health Research. The 10/90 report on health research 2001-2002.

[10] Vianna CM, Caetano R, Ortega JA, Façanha LO, Mosegui GB, Siqueiraf M (2007). Flows of financial resources for health research and development in Brazil, 2000–2002. Bulletin of the World Health Organization.

[11] Why invest in surveillance?. http://www.who.int/ncd_surveillance/en/.

[12] Wildlife Conservation Society (2008, October 8). ‘Deadly Dozen’ reports diseases worsened by climate change. Science Daily.

[13] McMichael AJ, Woodruff RE, Hales S (2006). Climate change and human health: present and future risks. Lancet.

[14] World Health Day 2008: Protecting health from climate change. http://www.who.int/world-health-day/en/index.html.

[15] Pandve H (2007). Global warming: Need to sensitize general population. Indian J Occup Environ Med.

[16] Pandve HT (2008). Emerging public health issues due to climate change. Indian J Occup Environ Med.

